# Nerve Bundle Density and Expression of NGF and IL-1β Are Intra-Individually Heterogenous in Subtypes of Endometriosis

**DOI:** 10.3390/biom14050583

**Published:** 2024-05-15

**Authors:** Mahfuza Sreya, Dwayne R. Tucker, Jennifer Yi, Fahad T. Alotaibi, Anna F. Lee, Heather Noga, Paul J. Yong

**Affiliations:** 1 Department of Obstetrics and Gynaecology, University of British Columbia, Vancouver, BC V6T 2A1, Canada; 2 Women’s Health Research Institute, Vancouver, BC V6H 2N9, Canada; 3 Department of Physiology, College of Medicine, Imam Mohammad Ibn Saud Islamic University (IMSIU), Riyadh 13317, Saudi Arabia; 4 Department of Pathology and Laboratory Medicine, University of British Columbia, Vancouver, BC V6T 1Z7, Canada; 5 Centre for Pelvic Pain and Endometriosis, BC Women’s Hospital & Health Centre, Vancouver, BC V6H 3N1, Canada

**Keywords:** endometriosis, nerve growth factor, interleukin-1, neuroproliferation, chronic pelvic pain, dyspareunia

## Abstract

Endometriosis is a gynecological disorder associated with local inflammation and neuroproliferation. Increased nerve bundle density has been attributed to increased expression of nerve growth factor (NGF) and interleukin–1β (IL-1β). Immunohistochemical analysis was carried out on 12 patients presenting with all three anatomic subtypes of endometriosis (deep, superficial peritoneal, endometrioma) at surgery, with at least two surgically excised subtypes available for analysis. Immunolocalization for nerve bundle density around endometriosis using protein gene product 9.5 (PGP9.5), as well as NGF and IL-1β histoscores in endometriosis epithelium/stroma, was performed to evaluate differences in scores between lesions and anatomic subtypes per patient. Intra-individual heterogeneity in scores across lesions was assessed using the coefficient of variation (CV). The degree of score variability between subtypes was evaluated using the percentage difference between mean scores from one subtype to another subtype for each marker. PGP9.5 nerve bundle density was heterogenous across multiple subtypes of endometriosis, ranging from 50.0% to 173.2%, where most patients (8/12) showed CV ≥ 100%. The percentage difference in scores showed that PGP9.5 nerve bundle density and NGF and IL-1β expression were heterogenous between anatomic subtypes within the same patient. Based on these observations of intra-individual heterogeneity, we conclude that markers of neuroproliferation in endometriosis should be stratified by anatomic subtype in future studies of clinical correlation.

## 1. Introduction

Endometriosis is a common gynecological disorder characterized by the presence of endometrial-like glandular epithelial cells (GECs) and stromal cells (SCs) outside of the uterus, which may present with symptoms of dysmenorrhea, chronic pelvic pain, dyspareunia, and infertility in addition to other co-morbidities [1,2]. Endometriosis can be classified into three anatomic subtypes: deep endometriosis (DE), superficial peritoneal (SUP), and endometrioma (OMA). The severity of endometriosis can be described by the #Enzian classification system and can also be described by the revised American Society for Reproductive Medicine classification into four stages based on size of the lesion, anatomic location, and extent of adhesion: Stage I: mild; Stage II: moderate; Stage III: severe; and Stage IV: extensive [3,4,5].

The extent of disease and severity of pain does not seem to be correlated in endometriosis, and the stage may not provide the most robust explanation of the patients’ pain symptoms [6]. The concept of neuroproliferation in association with dyspareunia in endometriosis has been described as a quantitative increase in nerve fiber bundles around the endometriotic lesion, which may amplify pain signaling to the central nervous system when contacted [7]. The nerve growth factor family of neurotrophins have been implicated in increased local neuroproliferation and pain, specifically with nerve growth factor (NGF) as the primary neurotrophin of interest [8,9,10,11,12,13,14,15,16,17].

A pathway of local neuroproliferation may be triggered or amplified by simultaneous local inflammation. The inflammatory cytokine interleukin-1β, (IL-1β) has been proposed to directly influence NGF and BDNF expression in endometriotic SC, which in turn stimulates nerve bundle growth identified using the pan-neuronal specific ubiquitin carboxyl-terminal hydrolase isoenzyme, protein-gene-product 9.5 (PGP9.5) [16,18,19,20,21,22,23]. Local inflammation may be further amplified by the resulting neuroproliferation, leading to a positive feedback loop between neuroproliferation and pain [7]. Recently, studies have begun to explore the IL-1β-NGF pathway to pain in relation to endometriosis phenotypes [18]. Previously, we proposed a model of endometriosis-related pain phenotyping based on potential peripheral pain mechanisms, specifically local inflammation and neuroproliferation [5,6]. However, studies have produced variable results in correlating local inflammation and neuroproliferation with anatomic subtype and pain symptoms [10,11,14,24].

A factor that may confound the clinical correlation of local neuroproliferation and inflammation in endometriosis is the issue of multiple anatomic subtypes and multiple lesions per subtype in a single patient. In this study, we describe twelve patients undergoing surgery for endometriosis with additional immunohistochemical testing for NGF, IL-1β, and nerve bundle density using PGP9.5. The objective of this study is to characterize the intra-individual heterogeneity of these markers amongst patients with endometriosis, which may guide future clinical correlation studies for neuroproliferation in endometriosis.

## 2. Materials and Methods

### 2.1. Study Description

Twelve patients were selected from a cohort of 122 patients from the BC Centre for Pelvic Pain and Endometriosis that had previously been described in an earlier study that included the methodology for PGP9.5 nerve bundle density (Table 1) [25]. This study was approved by the Research Ethics Board of the University of British Columbia (ENDOONC study; REB H11-00536 and H14-03040). Clinical data were collected as part of a linked prospective registry at the center (EPPIC registry, https://clinicaltrials.gov #NCT02911090 (accessed on 6 October 2023), REB H11-02882 and H16-00264). Informed consent was received from all study subjects for the ENDOONC study and EPPIC data registry.

**Figure 1:**
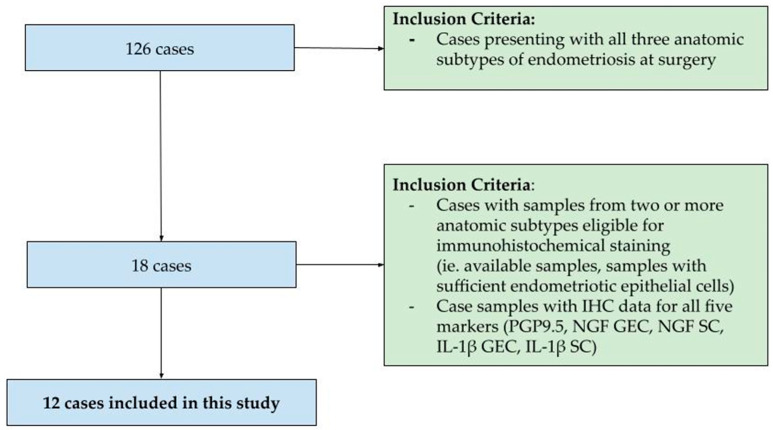
Inclusion/exclusion criteria for intra-individual neuroproliferative immunohistochemistry.

The 12 patients in this study were surgically diagnosed with all three anatomic subtypes at the time of an index surgery that took place between 1 December 2013 and 31 December 2017. Formalin-fixed paraffin-embedded tissue specimens from sites of suspected endometriosis were preserved for pathology at the time of surgery. Samples were screened for tissue quality (i.e., adequate endometriotic glandular epithelial cells (GECs) and stromal cells (SCs)) using hematoxylin and eosin staining prior to selection for analysis. Cases were selected based on the availability of samples from at least two anatomic subtypes eligible for immunolocalization. These 12 patients were selected for a demonstration of intra-individual variation in NGF, IL-1β, and nerve bundle density by PGP9.5 (Figure 1).

### 2.2. Immunohistochemistry

Immunohistochemistry (IHC) and scoring for PGP9.5, NGF, and IL-1β were performed as previously described [17,18,24]. Briefly, IHC was performed on 4 μm thick formalin-fixed paraffin-embedded sections. Staining for pan-neuronal marker PGP9.5 was conducted using mouse anti-human PGP9.5 antibody (NCL-L-PGP9.5, Leica Biosystems, Wetzlar, Germany, dilution 1:200). Staining for NGF and IL-1β was conducted using rabbit anti-human proNGF antibody (ab52918, Abcam, Cambridge, UK, dilution 1:400) and rabbit anti-human IL-1β antibody (ab2105, Abcam, Cambridge, UK, dilution 1:100), respectively. Diaminobenzidine secondary antibody staining was performed on the automated Dako Omnis platform (Dako, Agilent, Santa Clara, CA, USA).

**Figure 2:**
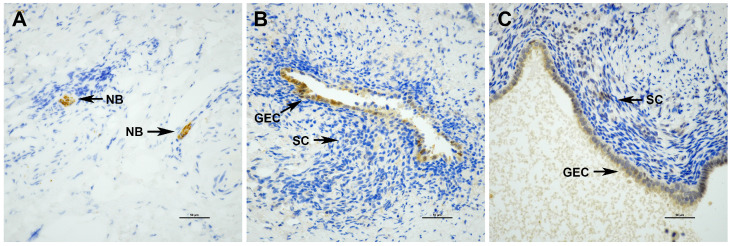
An example of immunolocalization of (**A**) PGP9.5 nerve bundle density, (**B**) NGF, and (**C**) IL-1β in the left uterosacral DE lesion of Patient 4. NB: nerve bundle; GEC: glandular epithelial cells; SC: stromal cells. Magnification, 400×; bar = 50 μm.

PGP9.5 nerve bundle density was calculated as the number of PGP9.5-positive nerve bundles at a 200× magnification divided by the total number of high-powered fields observed (fiber bundles/HPF) (Figure 2). Histoscores were calculated for NGF and IL-1β in HPF fields of endometriosis at a 200× magnification as previously described [26]. Briefly, the intensity of NGF and IL-1β immunostaining was categorized (0 = negative; 1 = weak; 2 = moderate; 3 = strong) in glandular epithelial cells and stromal cells, and the percentage of stained cells in each of the 4 categories was visually estimated for the specific cell type (epithelial or stromal). The histoscore was then calculated for each cell type in each of the three random fields as follows:

$$Histoscore=\;\left(0\times \%\;of\;negatively\;stained\;cells\right)+\;\left(1\times \%\;of\;weakly\;stained\;cells\right)+\;\left(2\times \%\;of\;moderately\;stained\;cells\right)+\;\left(3\times \%\;of\;strongly\;stained\;cells\right)$$

Histoscores of each sample were calculated as the mean of the histoscores from the three random scored fields.

### 2.3. Statistical Analysis

Overall, five variables were considered: PGP9.5 nerve bundle density around endometriosis, NGF histoscores in endometriosis GEC and SC, and IL-1β histoscores in endometriosis GEC and SC. These variables were examined across anatomic subtypes within each patient and presented as the median and interquartile range, with intra-individual heterogeneity displayed by the coefficient of variation (mean divided by standard deviation). As well, PGP9.5 nerve bundle density and NGF and IL-1β histoscores, were compared between anatomic subtypes within the same patient. The relative magnitude of difference between subtypes within each patient for each marker was calculated as follows:

$$Percentage\;Difference\;\left(PD\right)=\frac{\left({Histoscore}_{Subtype\;1}-{Histoscore}_{Subtype\;2}\right)}{{Histoscore}_{avg}}\times 100\%$$

For patients with more than one lesion for an anatomic subtype (e.g., DE), the average nerve density and histoscore were used. The analysis was performed using Microsoft Excel 2019 (Microsoft Corporation, Redmond, WA, USA), IBM SPSS 28.0 (IBM Corporation, Armonk, NY, USA) and GraphPad Prism 9 software (GraphPad Software, San Diego, CA, USA).

## 3. Results

### 3.1. Intra-Individual Variation in Nerve Bundle Density, NGF, and IL-1β

Immunohistochemistry and analysis for protein gene product 9.5 (PGP 9.5) nerve bundle density, NGF GEC, NGF SC, IL-1β GEC, and IL-1β SC were performed in the following lesions across the twelve patients (Table 2, Figure S1).

The coefficients of variation (CV) of each marker assessed the heterogeneity in scores between the lesions (across anatomic subtypes) of each patient (Table 2). Most patients in this cohort (Patients 1–3, 5, 7, 8, 10, and 12) had CVs greater than 100% for PGP9.5 nerve bundle density (101.9–173.2%), indicating that the standard deviations were greater than the mean score or that the marker had a higher degree of variability (Table 2). However, intra-individual heterogeneity was not similarly prevalent in markers of local neuroproliferation, with fewer patients presenting with a high variability of expression between lesions, for example, NGF GEC (Patient 11: 104.6%), NGF SC (Patient 1: 106.7%; Patient 8: 139.1%), IL-1β GEC (Patient 1: 117.9%), or IL-1β SC (Patient 1: 141.4%; Patient 8: 173.2%) (Table 2).

### 3.2. Nerve Bundle Density and Expression of NGF and IL-1β Vary between Anatomic Subtypes

The mean difference in scores between anatomic subtypes was used to evaluate the degree of variation for PGP9.5 nerve bundle density and for NGF and IL-1β expression. The percentage difference (PD) in scores was used to determine the relative magnitude of the differences in mean scores between anatomic subtypes.

#### 3.2.1. Patients with DE and SUP

**Figure 3:**
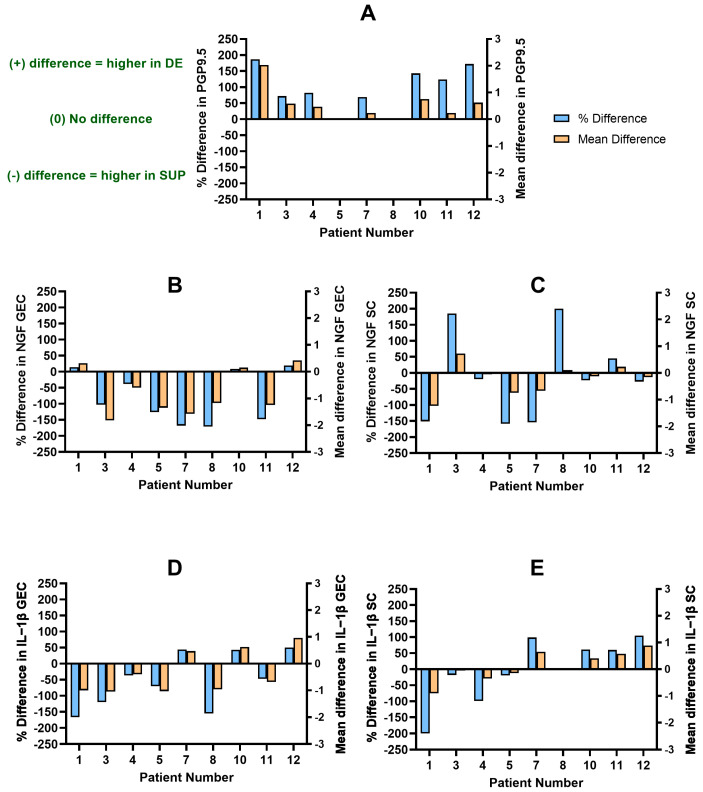
The difference in PGP9.5 nerve bundle density and IL-1β and NGF expression between DE and SUP per patient. A difference of >0% or >0 indicates a higher score in DE; a difference of <0% or <0 indicates a higher score in SUP. (**A**) Distribution of difference in PGP9.5 nerve bundle density. (**B**) Distribution of difference in NGF GEC histoscore. (**C**) Distribution of difference in NGF SC histoscore. (**D**) Distribution of difference in IL-1β GEC histoscore. (**E**) Distribution of difference in IL-1β SC histoscore. PGP9.5: protein gene product 9.5; NGF: nerve growth factor; IL-1β: interleukin-1β; GEC: glandular epithelial cells; SC: stromal cells.

Nine patients had available DE and SUP lesions for analysis and comparison (Table 3, Figure 3A; Patients 1, 3, 4, 5, 7, 8, 10, 11, and 12). Seven patients had a higher nerve bundle density across the DE lesions compared to the SUP lesions (PD: +69% to +187%) (Figure 3A; Patients 1, 3, 4, 7, 10, 11, and 12). However, the differences in NGF and IL-1β expression between DE and SUP lesions varied greatly. As expected, Patients 10 and 12 showed overall higher scores in the DE lesions for NGF GEC (PD: +9% to +19%), IL-1β GEC (PD: +43% to +50%), and IL-1β SC (PD: +61% to +105%), whereas only NGF SC (PD: −27% to −23%) was higher in the SUP lesion (Figure 3B–E; Patients 10, 12). However, Patients 1, 3, and 4 showed overall higher scores in the SUP lesions for NGF GEC (PD: −103% to +14%), NGF SC (PD: −151% to +185%), IL-1β GEC (PD: −167% to −37%), and IL-1β SC (PD: −200% to −18%) (Figure 3B–E; Patients 1, 3, and 4).

Two patients with higher PGP9.5 in DE had a higher NGF and IL-1β expression in either the SUP or DE lesions (Patient 7 and 11). Patient 7 had a higher expression of NGF GEC (PD: −169%) and NGF SC (PD: −154%) in the SUP lesion and a higher expression of IL-1β GEC (PD: +44%) and IL-1β SC (PD: +99%) in the DE lesion (Figure 3B–E; Patient 7). In contrast, Patient 11 showed a higher expression of NGF GEC (PD: −148%) and IL-1β GEC (PD: −47%) in the SUP lesion and a high expression of NGF SC (PD: +45%) and IL-1β SC (PD: +60%) in the DE lesion (Figure 3B–E; Patient 11).

Patients 5 and 8 had no difference in PGP9.5 nerve bundle density between their DE and SUP lesions (PD: 0%), yet both showed patterns of higher NGF and IL-1β expression in either subtype (Figure 3A; Patients 5 and 8). Patient 5 had an overall higher expression in the SUP lesion for NGF GEC (PD: −126%), NGF SC (PD: −20%), IL-1β GEC (PD: −70%), and IL-1β SC (PD: −99%) compared to the DE lesion (Figure 3B–E; Patient 5). Patient 8 showed a higher expression in the DE lesion for NGF SC (PD: +200%) but a higher expression in the SUP lesion for NGF GEC (PD: −171%) and IL-1β GEC (PD: −155%) (Figure 3B–D; Patient 8). There was no difference in IL-1β SC expression (PD: 0%) between DE and SUP in Patient 8 (Figure 3E; Patient 8).

#### 3.2.2. Patients with DE and OMA

**Figure 4:**
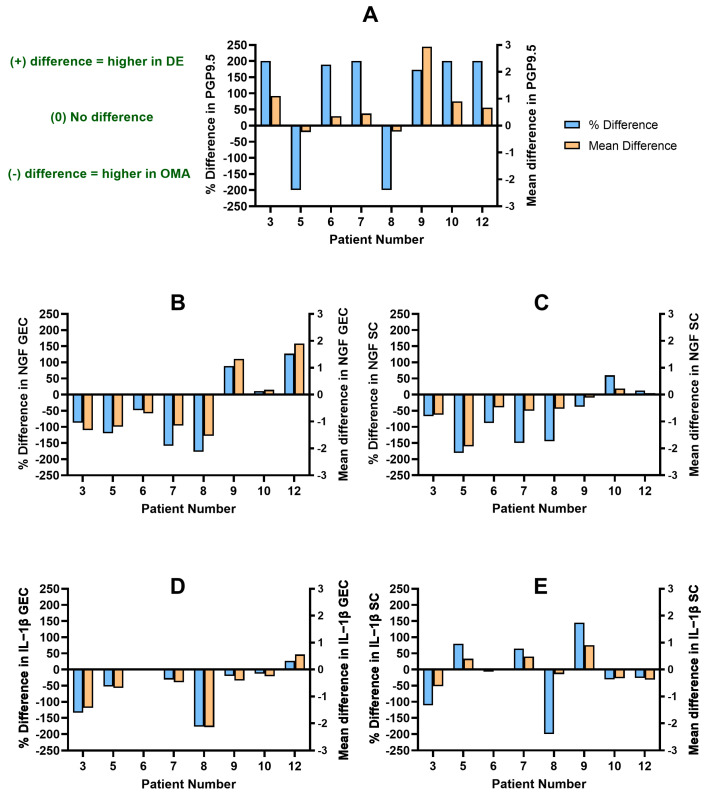
The difference in PGP9.5 nerve bundle density and IL-1β and NGF expression between DE and OMA per patient. A difference of >0% or >0 indicates a higher score in DE; a difference of <0% or <0 indicates a higher score in OMA. (**A**) Distribution of difference in PGP9.5 nerve bundle density. (**B**) Distribution of difference in NGF GEC histoscore. (**C**) Distribution of difference in NGF SC histoscore. (**D**) Distribution of difference in IL-1β GEC histoscore. (**E**) Distribution of difference in IL-1β SC histoscore. PGP9.5: protein gene product 9.5; NGF: nerve growth factor; IL-1β: interleukin-1β; GEC: glandular epithelial cells; SC: stromal cells.

Eight patients had available DE and OMA lesions for analysis (Table 3, Figure 4; Patients 3, 5, 6, 7, 8, 9, 10, and 12). Six patients had a higher PGP9.5 nerve bundle density in DE lesions compared to the OMA (PD: +173% to +200%) (Figure 4A; Patients 3, 6, 7, 9, 10, and 12). Only Patient 12 showed overall higher scores in the DE lesion for NGF GEC (PD: +127%), NGF SC (PD: +13%), and IL-1β GEC (PD: +27%), whereas IL-1β SC (PD: −26%) was higher in the OMA (Figure 4B–E; Patient 12). Despite a higher nerve density in DE among the rest, three patients showed overall higher scores in the OMA lesion for NGF GEC (PD: −159% to −87%), NGF SC (PD: −150% to −67%), IL-1β GEC (PD: −134% to −1%), and IL-1β SC (PD: −111% to +65%) (Figure 4B–E, Patients 3, 6, and 7). Patients 9 and 10 showed more variability in marker expression, as their scores for NGF GEC (PD: +11% to +89%), NGF SC (PD: −38% to +60%), IL-1β GEC (PD: −20% to −13%), and IL-1β SC (PD: −31% to +145%) were higher in either the DE or OMA (Figure 4B–E; Patients 9 and 10).

In contrast, two patients showed higher PGP9.5 scores in the OMA when compared to the DE lesion with an expected NGF and IL-1β expression between the two subtypes (PD: −200%) (Figure 4A; Patients 5 and 8). The patients scored overall higher in the OMA for NGF GEC (PD: −177% to −120%), NGF SC (PD: −181% to −145%), and IL-1β GEC (PD: −177% to −53%) compared to their DE (Figure 4B–D; Patients 5 and 8). Only Patient 8 had a higher score for IL-1β SC (PD: −200%) in the OMA as well (Figure 4E; Patient 8).

#### 3.2.3. Patients with SUP and OMA

**Figure 5:**
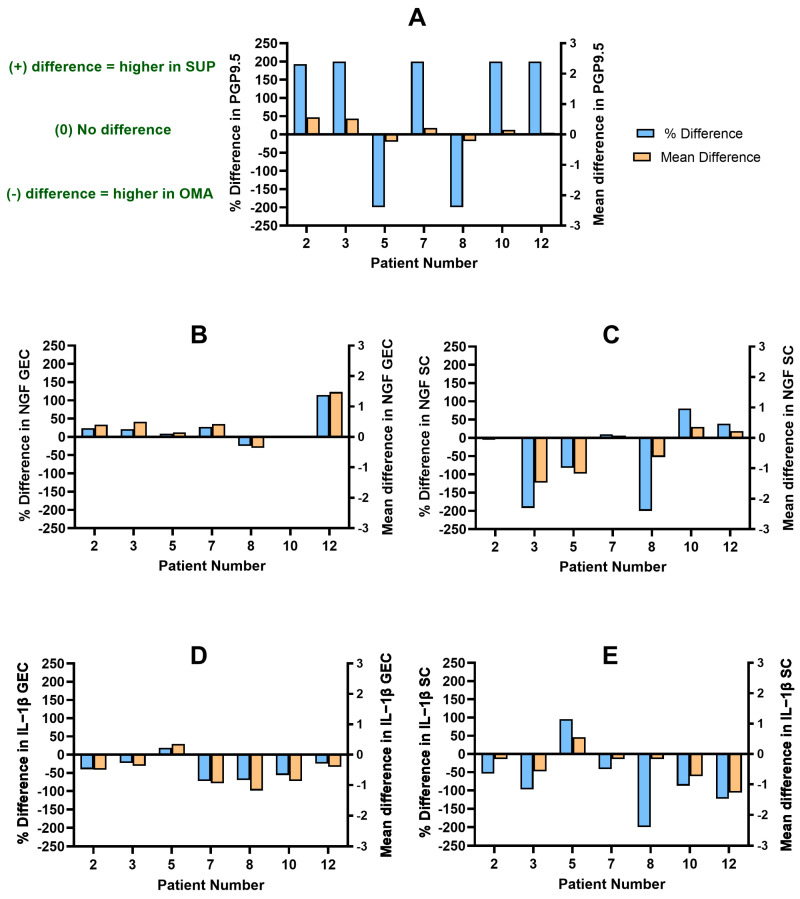
The difference in PGP9.5 nerve bundle density and IL-1β and NGF expression between SUP and OMA per patient. A difference of >0% or >0 indicates a higher score in SUP; a difference of <0% or <0 indicates a higher score in OMA. (**A**) Distribution of difference in PGP9.5 histoscore. (**B**) Distribution of difference in NGF GEC histoscore. (**C**) Distribution of difference in NGF SC histoscore. (**D**) Distribution of difference in IL-1β GEC histoscore. (**E**) Distribution of difference in IL-1β SC histoscore. PGP9.5: protein gene product 9.5; NGF: nerve growth factor; IL-1β: interleukin-1β; GEC: glandular epithelial cells; SC: stromal cells.

Seven patients had available SUP and OMA lesions for analysis (Table 3, Figure 5; Patients 2, 3, 5, 7, 8, 10, and 12). Of the seven, five patients had higher PGP9.5 nerve bundle density scores in the SUP lesion compared to the OMA (PD: +193% to +200%) (Figure 5A; Patients 2, 3, 7, 10, and 12). However, these patients had inconsistent NGF and IL-1β expressions between the two anatomic subtypes. The scores for NGF GEC (PD: +2% to +115%) and NGF SC (PD: +10 to +80%) were higher in the SUP lesion, while the scores for IL-1β GEC (PD: −72% to −25%) and IL-1β SC (PD: −123% to −41%) were higher in the OMA in Patients 7, 10, and 12 (Figure 5B–E; Patients 7, 10, and 12). As well, two patients had overall higher scores within the OMA lesion for NGF SC (PD: −192% to −5%), IL-1β GEC (PD: −40% to −23%), and IL-1β SC (PD: −97% to −54%), with only the NGF GEC scores (PD: +21% to +24%) being higher in the SUP lesion (Figure 5B–E; Patients 2 and 3).

Two patients had a higher PGP9.5 nerve bundle density in the OMA compared to the SUP lesion, yet NGF and IL-1β expression was not consistent between the two individuals either (Figure 5A; Patients 5 and 8). As expected by the PGP9.5 score, Patient 8 had overall higher scores in the OMA for NGF GEC (PD: −25%), NGF SC (PD: −200%), IL-1β GEC (PD: −70%), and IL-1β SC (PD: −200%) (Figure 5B–E; Patient 8). However, Patient 5 presented with overall higher scores in the SUP lesion for NGF GEC (PD: +9%), IL-1β GEC (PD: +19%), and IL-1β SC (PD: +96%), where only NGF SC (PD: −82%) was higher in the OMA (Figure 5B–E; Patient 5).

## 4. Discussion

In this study, we present the intra-individual heterogeneity in PGP9.5 nerve bundle density and associated biomarkers of neuroproliferation (NGF, IL-1β), amongst patients who had all three anatomic subtypes of endometriosis at surgery and where at least two subtypes were available for analysis. Coefficients of variation (CV) for PGP9.5 nerve bundle density and histoscores for the other biomarkers were examined in each patient. Most CVs were less than 100% (i.e., standard deviation less than mean), but the CV was highest for PGP9.5 nerve bundle density, being at times higher than 100%. For PGP9.5 nerve bundle density and the other biomarker histoscores, there were wide differences in terms of the relative expression levels between anatomic subtypes within the same patient.

The lower CV for NGF GEC, NGF SC, IL-1β GEC, and IL-1β SC (Table 3) could suggest that it may be plausible to sample one lesion in a patient as a reflection of expression for the patient. However, there were differences in level of expression between each anatomic subtypes in the same patient (Figure 2, Figure 3 and Figure 4). Therefore, it is recommended that each anatomic subtype be sampled separately within a patient, as expression in one subtype cannot be assumed to reflect expression in another subtype.

These observations may guide future studies attempting a clinical correlation of PGP9.5 nerve bundle density and associated neuroproliferative biomarkers. In a patient with multiple anatomic subtypes present, it is not possible to correlate one anatomic subtype to pain symptoms. Instead, all available anatomic subtypes should be sampled per patient. For statistical analyses, we propose that correlations with pain severity be carried out by anatomic subtype. For example, in a cohort, the PGP9.5 nerve bundle density amongst DE lesions in the cohort can be analyzed for an association with deep dyspareunia; then, the PGP9.5 nerve bundle density amongst SUP lesions and the OMA lesions can be studied separately for associations with deep dyspareunia. This would reflect three overlapping non-mutually exclusive sub-cohorts within the total cohort, since each patient can have more than one anatomic subtype.

Another observation in this study is the complex relationship between PGP9.5 nerve bundle density and NGF and IL-1β histoscores in each patient. While these variables have been found to be correlated in prior studies [11,18,19], there remains irregularity with certain biomarkers being higher in one subtype and other biomarkers being higher in another subtype, within the same patient. In other words, patterns observed for NGF cannot be extrapolated to IL-1β, and vice versa. Therefore, we recommend that each biomarker be examined separately in future studies. As well, there are multiple other factors to be involved, including other cells in the endometriosis microenvironment (e.g., mast cells) and other neuroproliferative factors (e.g., BDNF and Trk receptors) that can confound these correlations [14,27].

A strength of this study is an in-depth look at the intra-individual variation in markers of local neuroproliferation, including across anatomic subtypes, which would not be possible in studies that only sample one lesion or anatomic subtype per patient [10,12,13,14,15,19,21,28]. The limitations are the sample size, such that descriptions are provided but statistical analyses of observed trends and correlations to patient-reported pain scores were not possible. We did not have sufficient cases with multiple lesions of each subtype (e.g., two OMAs) and thus could not examine the issue of heterogeneity within a single subtype in the same patient.

Further research will involve the clinical correlation of PGP9.5 nerve bundle density and associated biomarkers with an adjustment for hormonal treatment. Given that local neuroproliferation is just one of multiple pain generators in endometriosis from peripheral to central [7], this type of analysis will be complex. It will likely be necessary to control for pain comorbidities that are common in endometriosis, such as visceral pain conditions (irritable bowel syndrome and painful bladder syndrome) and somatic pain conditions (abdominal wall myofascial trigger points and pelvic floor myalgia) [29]. As such, studies will need to be standardized with rigorous pain phenotyping, and an adequate sample size will be required to control for potential confounders.

## 5. Conclusions

This case report highlights the intra-individual heterogeneity in nerve bundle density and NGF and IL-1β expression between anatomic subtypes of endometriosis in patients with greater disease burden. These observations should guide future studies that correlate these factors with clinical presentation.

## Figures and Tables

**Table 1:** Patient and sample characteristics describing the findings at intake and at the index surgery. * Deep dyspareunia within 3 months of index surgery: *N* = 11.

Variable	Median (IQR) or No. of Patients (%) *N* = 12
Patient characteristics	
Age	34.5 (30.25–36.75)
Parity	
Parous	3 (25.0%)
Nulliparous	9 (75.0%)
Ethnicity	
Caucasian only	9 (75.0%)
Other ethnicity	3 (25.0%)
Hormone Use	
Yes	7 (58.3%)
No	5 (41.7%)
Cul-de-sac tenderness	
Yes	9 (75.0%)
No	3 (25.0%)
Pelvic floor or bladder tenderness	
Yes	4 (33.3%)
No	8 (66.7%)
Findings at index surgery	
rASRM surgical staging	
III	5 (41.7%)
IV	6 (50.0%)
Missing	1 (8.3%)
Cul-de-sac obliteration	
Complete	3 (25.0%)
Partial	2 (16.7%)
None	7 (58.3%)
Pain Scores (0–10 scale)	
Deep dyspareunia within 3 months *	8.0 (5.0–9.0)
Dyschezia within 3 months	7.0 (4.0–9.0)

**Table 2:** Anatomic subtype and location of endometriosis selected for immunohistochemistry in patients presenting with all three anatomic subtypes. Cells marked with “N/A” represent samples which were unavailable or had insufficient endometriosis for analysis. R: right; L: left; ns: laterality not specified; DE: deep endometriosis; SUP: superficial peritoneal endometriosis; OMA: endometrioma.

Patient No.	DE	SUP	OMA
1	Ureter (L)	Ovary (ns)	N/A
2	N/A	Posterior cul-de-sac (L)	Ovary (ns)
3	Appendix	Uterosacral ligament (L)	Ovary (R)
4	Uterosacral ligament (L)	Pelvic sidewall (R)	N/A
5	Uterosacral ligament (L)	Fallopian tube (ns)	Ovary (R)
6	Uterosacral ligament (R), Uterosacral ligament (L), Posterior cul-de-sac	N/A	Ovary (R)
7	Uterosacral ligament (L)	Posterior cul-de-sac	Ovary (L)
8	Anterior cul-de-sac	Anterior cul-de-sac	Ovary (L)
9	Appendix, Fallopian tube (R), Posterior cervix, Rectum, Vagina	N/A	Ovary (R)
10	Periureteric (L)	Fallopian tube (L)	Ovary (L)
11	Pelvic sidewall (R)	Pelvic sidewall (L)	N/A
12	Bladder (L)	Pelvic sidewall (R)	Ovary (L)

**Table 3:** Nerve bundle density by PGP9.5 and histoscores of NGF and IL-1β across all sampled lesions per patient. PGP9.5: protein gene product 9.5; NGF: nerve growth factor; IL-1β: interleukin-1β; NBD: nerve bundle density; GEC: glandular epithelial cells; SC: stromal cells; IQR: interquartile range; CV: coefficient of variation.

	PGP9.5 NBD		NGF GEC		NGF SC		IL-1β GEC		IL-1β SC	
Patient No.	Median (IQR)	CV	Median (IQR)	CV	Median (IQR)	CV	Median (IQR)	CV	Median (IQR)	CV
1	1.1 (0.1–2.1)	132.3%	2.3 (2.1–2.5)	9.9%	0.8 (0.2–1.4)	106.7%	0.6 (0.1–1.1)	117.9%	0.5 (0.0–0.9)	141.4%
2	0.3 (0.0–0.6)	136.6%	1.7 (1.5–1.9)	16.6%	0.6 (0.6–0.6)	3.5%	1.3 (1.0–1.5)	28.3%	0.3 (0.2–0.4)	38.2%
3	0.5 (0.0–1.1)	101.9%	2.2 (0.9–2.7)	49.6%	0.8 (0.0–1.5)	96.7%	1.4 (0.4–1.8)	62.8%	0.3 (0.3–0.9)	72.8%
4	0.6 (0.3–0.8)	57.8%	1.6 (1.3–1.9)	26.9%	0.3 (0.2–0.3)	14.4%	1.1 (0.9–1.3)	26.4%	0.4 (0.2–0.5)	69.7%
5	0.0 (0.0–0.2)	173.2%	1.6 (0.4–1.8)	59.2%	0.9 (0.1–2.0)	97.9%	1.6 (1.0–2.0)	34.5%	0.7 (0.3–0.9)	46.1%
6	0.3 (0.1–0.4)	70.7%	1.3 (0.8–1.7)	35.9%	0.4 (0.2–0.7)	59.6%	1.8 (1.3–2.0)	20.6%	0.5 (0.3–0.7)	43.4%
7	0.2 (0.0–0.5)	100.8%	1.3 (0.2–1.7)	77.1%	0.7 (0.1–0.8)	70.4%	1.3 (0.8–1.8)	36.2%	0.5 (0.3–1.0)	55.9%
8	0.0 (0.0–0.2)	173.2%	1.3 (0.1–1.6)	79.9%	0.1 (0.0–0.6)	139.1%	1.1 (0.1–2.3)	91.4%	0.0 (0.0–0.2)	173.2%
9	3.2 (1.6–3.6)	50.0%	2.0 (1.6–2.4)	32.3%	0.2 (0.1–0.4)	56.1%	2.0 (1.5–2.4)	33.6%	1.1 (0.2–1.5)	71.7%
10	0.2 (0.0–0.9)	137.8%	1.6 (1.6–1.8)	5.9%	0.5 (0.3–0.6)	39.1%	1.8 (1.1–2.0)	27.5%	0.9 (0.5–1.2)	43.1%
11	0.2 (0.1–0.3)	87.9%	0.9 (0.2–1.5)	104.6%	0.5 (0.4–0.6)	31.6%	1.4 (1.1–1.8)	33.4%	1.0 (0.7–1.3)	42.7%
12	0.1 (0.0–0.7)	155.5%	2.0 (0.6–2.5)	59.5%	0.5 (0.5–0.7)	20.9%	1.8 (1.4–2.4)	25.6%	1.3 (0.4–1.7)	58.2%

## Data Availability

Anonymized raw data are not available as the participants of this study did not give written consent for their data to be shared publicly.

## Supplementary Materials

The following supporting information can be downloaded at: https://www.mdpi.com/article/10.3390/biom14050583/s1, Figure S1. Individual histoscores of PGP9.5, IL-1β, and NGF per anatomic location and anatomic subtype. (a) PGP9.5 nerve bundle density; (b) NGF GEC; (c) NGF SC; (d) IL-1β GEC; (e) IL-1β SC. CDS: cul-de-sac; USL: uterosacral ligament; R: right; L: left; ns: laterality not specified.
