# Supplementary figures and images for: Nerve Bundle Density and Expression of NGF and IL-1β Are Intra-Individually Heterogenous in Subtypes of Endometriosis
Source: Biomolecules. 2024 May 15;14(5):583. doi: 10.3390/biom14050583 (PMC11118880; doi:10.3390/biom14050583)

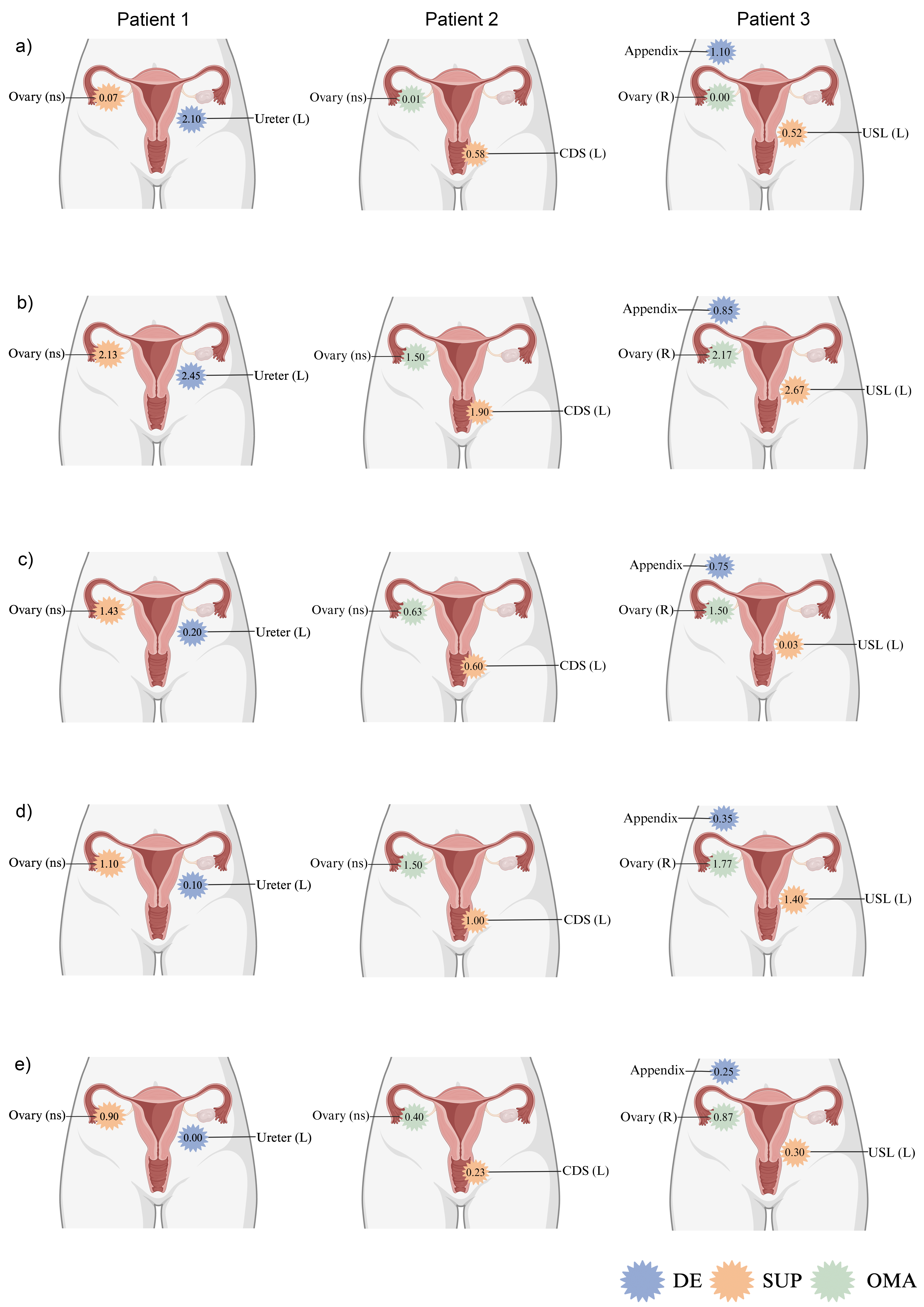

Supplement: Supplementary file 1 [file biomolecules-14-00583-s001.zip › Figure S1.1.tif]

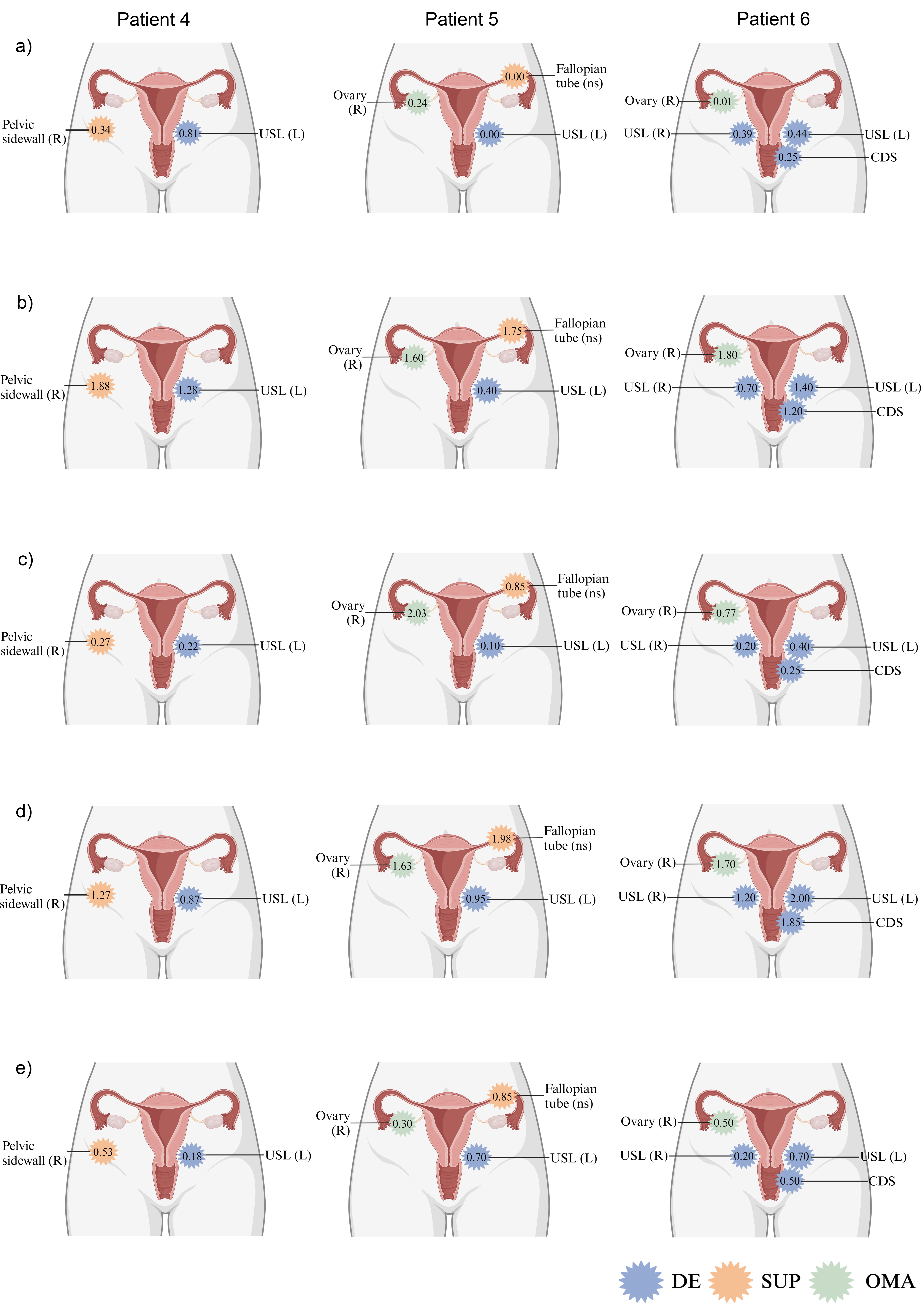

Supplement: Supplementary file 1 [file biomolecules-14-00583-s001.zip › Figure S1.2.tif]

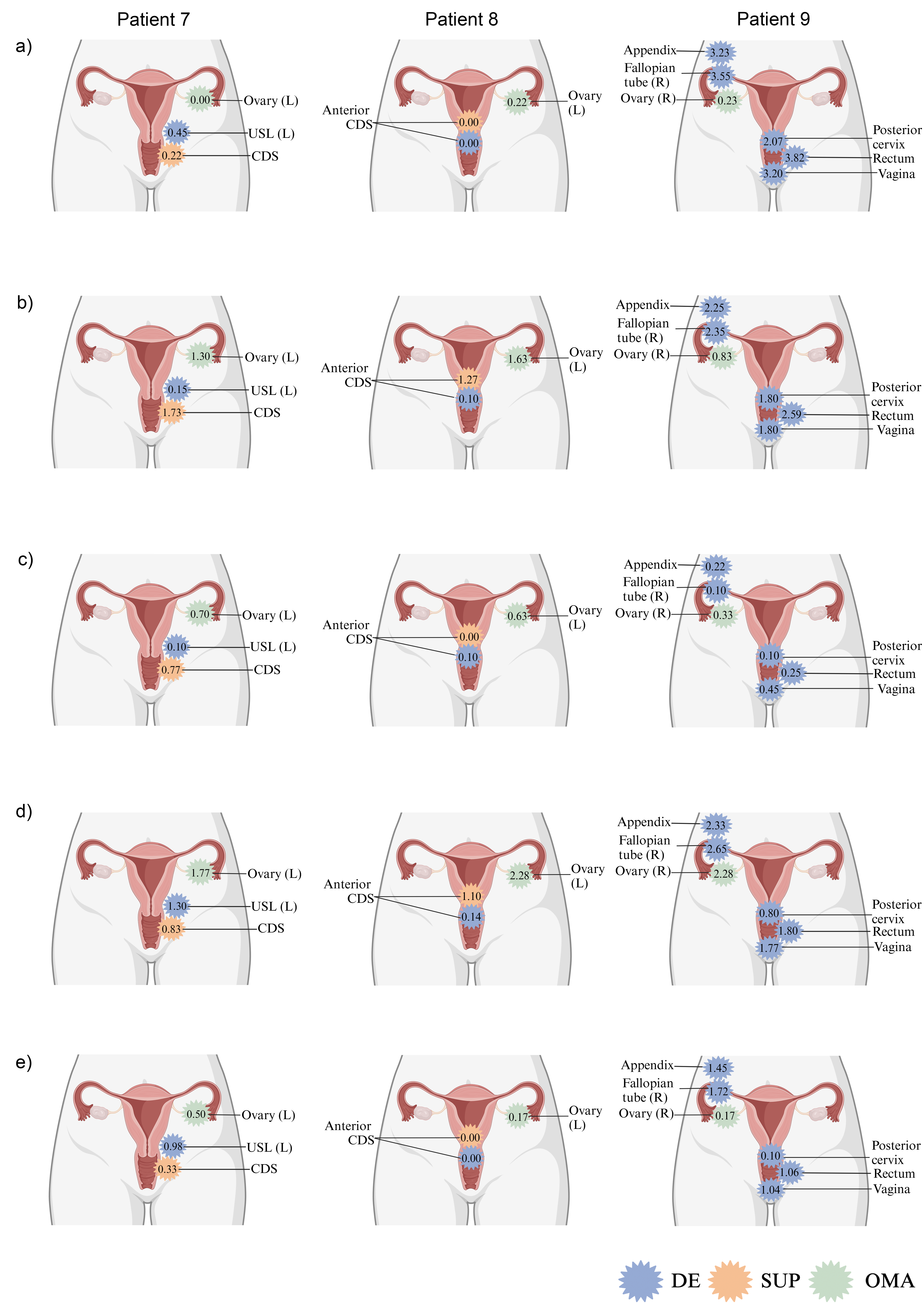

Supplement: Supplementary file 1 [file biomolecules-14-00583-s001.zip › Figure S1.3.tif]

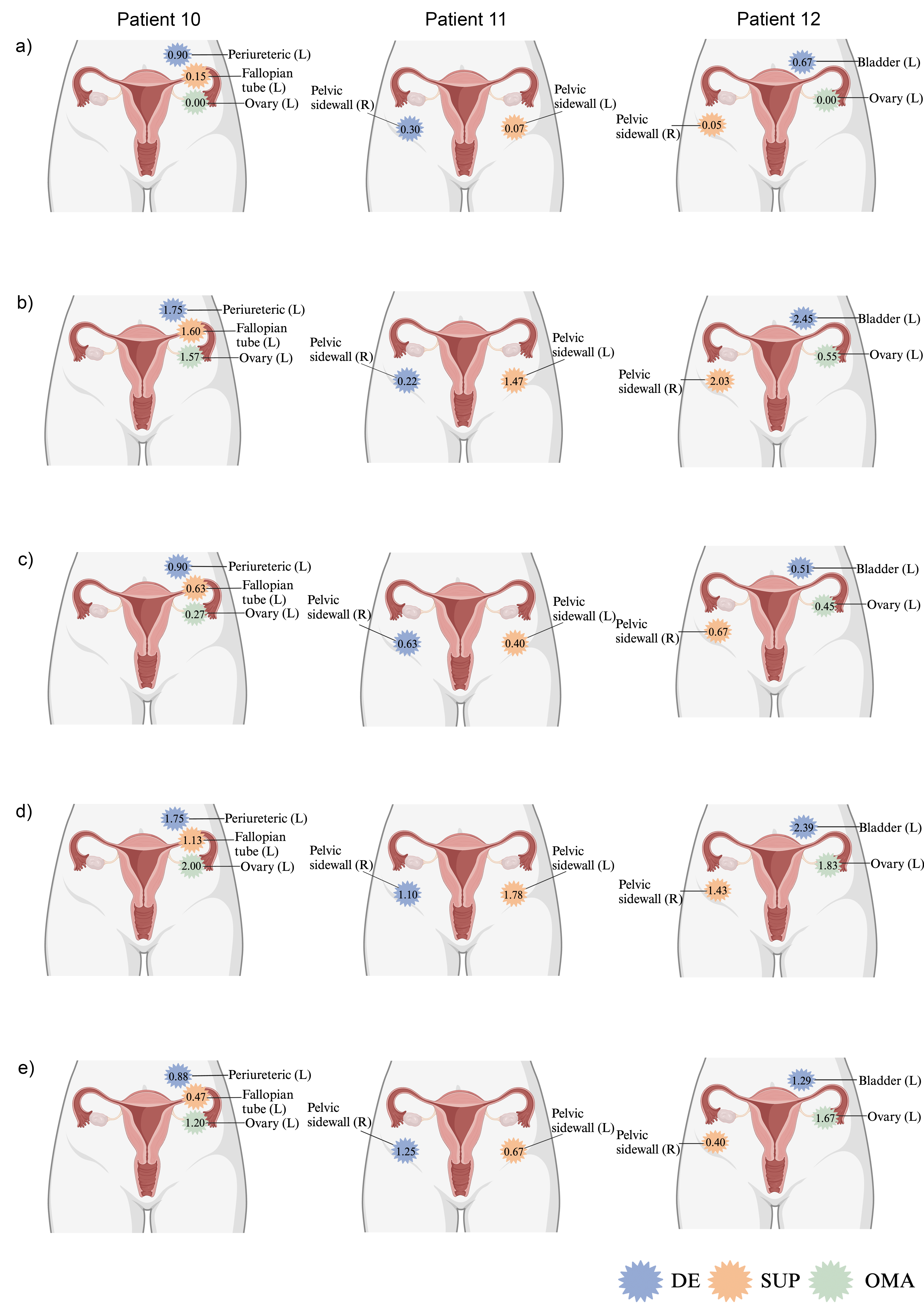

Supplement: Supplementary file 1 [file biomolecules-14-00583-s001.zip › Figure S1.4.tif]
